# Echoes of Home: The Impact of Acculturative Stress on Nigerian Students in Northern Cyprus, Investigating the Role of Loneliness and Social Support

**DOI:** 10.3390/bs15030289

**Published:** 2025-02-28

**Authors:** Yosra Jarrar, Gabriel E. Nweke

**Affiliations:** 1 Department of Communication and Information Studies, Mohammed Bin Rashid School for Communication, American University in Dubai, Dubai P.O. Box 28282, United Arab Emirates; 2 Department of Psychology, Faculty of Humanities, Girne American University, Girne 99138, Cyprus; nwekegabriel@gau.edu.tr

**Keywords:** acculturative stress, depression, loneliness, perceived social support, Nigeria international students

## Abstract

International students navigate a complex ecosystem influenced by various interrelated factors such as academic settings, family influences, social supports, and the process of cultural adjustment. While universities provide institutional support, the direct or indirect effect of family and social support network in the adaptation process and mental wellbeing is paramount. The interplay of acculturative stress, loneliness, perceived social support, and depression among international students warrants extensive study due to its profound impact on mental health and academic outcomes. This research aims to investigate the mediating role of loneliness and the moderating influence of perceived social support in the relationship between acculturative stress and depression among Nigerian university students in Northern Cyprus. Utilizing a cross-sectional quantitative research design, data were collected via a Google Survey from 392 Nigerian international students residing in Northern Cyprus. Participants completed self-report questionnaires assessing acculturative stress, loneliness, perceived social support, depression, and demographic details. Results indicate that acculturative stress directly influences depression, with loneliness partially mediating this relationship. Additionally, the impact of acculturative stress on loneliness and depression is significantly moderated by participants’ perceived social support. These findings underscore the importance of addressing acculturative stress and fostering social support networks to mitigate depressive symptoms among Nigerian students studying abroad. Further research and interventions aimed at enhancing social support mechanisms are imperative to promote the mental well-being of this population.

## 1. Introduction

With the advent of globalization, Northern Cyprus (TRNC) annually welcomes a significant number of international students pursuing their educational goals. While students from neighboring Turkey constitute most of the student body, Nigeria stands out as a leading contributor to international student enrollment, forming a substantial portion of the overall international student community. According to the Higher Education and Foreign Relations Office of TRNC, at the time of this study, universities in North Cyprus accommodated over 93,000 students, with approximately 29,600 being international students from over 100 different countries, representing roughly 30% of the total student population. Notably, students from Turkey, although comprising the largest share of the student body, are not classified as international students. Following closely in second place is Nigeria, accounting for approximately 26.7% of the total international student population (Ezel & Arasli, 2021).

The inclusion of Nigerian students, with their diverse cultural backgrounds and global outlooks, enriches the educational environment for everyone involved. Their presence offers a wealth of intellectual assets that enhance the available knowledge, resources, and skill sets at their universities. However, despite the undeniable benefits of multicultural experiences, several studies have underscored the significant acculturation stress faced by many international students as they transition from their native countries to their host nations (Nilsson et al., 2008).

Such stress is often magnified by unfamiliarity with the new culture and education system, as well as the distance from family, potentially leading to an increased risk of depression if not properly managed (M. H. Luz, 2020). Among the primary stressors affecting international students are language difficulties, academic pressure, financial issues, loneliness, social isolation, and experiences of discrimination (Koo et al., 2021; Lovell et al., 2015; Yan, 2020). To navigate through these hurdles, many international students draw upon the cultural strengths and practices from their home countries (Gebregergis, 2018). However, the cultural norms of the host country can sometimes conflict with those of their own, complicating their adjustment process.

Nigerian students often face heightened stress levels due to unique pressures, including the high value placed on academic success for family honor, a cultural emphasis on concealing emotions, and a deep-seated skepticism towards mental health services (Kukoyi et al., 2022; Aluh et al., 2018). For them, academic achievement is typically seen as crucial to their self-esteem and familial duty, making the prospect of failure especially daunting as it not only affects personal achievement but also societal expectations and familial honor (Toyin & Akoraro, 2009). Furthermore, in the “collectivist” Nigerian culture, there is a strong emphasis on maintaining a cohesive social framework, wherein controlling one’s emotions, particularly negative ones, is paramount for preserving the peace and harmony of relationships. Consequently, Nigerian international students may choose to internalize their stress rather than seeking support from individuals outside their cultural group, such as counselors, perceiving it as a violation of their cultural norms or as revealing vulnerability that could result in feelings of shame and guilt (Annamaria, 2014; Wilczewski et al., 2023).

The absence of a familiar support network, encompassing both family and friends, can profoundly affect these students, particularly if they have yet to establish comparable support systems in the host country. Despite its critical significance and growing relevance, there exists a noticeable research gap regarding the impact of acculturation stress on the mental well-being of Nigerian international students. Therefore, the objective of this study is to explore the intricate dynamics among acculturative stress, loneliness, and depression among Nigerian international students, while also examining the moderating influence of perceived social support.

### 1.1. Relations Between Acculturative Stress and Depression

Acculturation involves the psychological transformation individuals undergo as they engage in firsthand interactions with diverse cultures (Berry, 2005). For international students, adapting to a new language, cultural beliefs, and social norms presents significant challenges that can lead to heightened stress or anxiety (Bethel et al., 2020; Ma, 2017). This response to the disparities between one’s native culture and the predominant host culture is termed acculturative stress (Berry, 2006). It manifests in various stress-related behaviors, including feelings of estrangement, anxiety, marginalization, depression, increased physical symptoms related to stress, and identity crisis (Gebregergis, 2018). According to Berry (2006), acculturative stress encompasses emotional, physical, and interpersonal challenges encountered while acclimating to a different cultural or social setting. Research underscores a strong correlation between acculturative stress and depressive symptoms among student groups, highlighting the intricate relationship between cultural adjustment and mental well-being (Comer, 2015; Janssen-Kallenberg et al., 2017; Ma, 2017; Meghani & Harvey, 2016).

Depression, a significant mental health concern, encompasses symptoms such as diminished interest, feelings of sadness, reduced self-esteem, sleep disturbances, difficulty concentrating, guilt, and decreased energy, impacting cognition, emotions, and behaviors (Hosseinpur et al., 2023). These symptoms can persist and recur, hindering individuals’ ability to fulfill daily obligations (Gebregergis, 2018), affecting diverse demographics, including men, women, students, employees, teenagers, and particularly international students (Dhanoa et al., 2022). For international students, depression poses a considerable challenge, with acculturation stress strongly linked to its occurrence (D’Anna-Hernandez et al., 2015; Janssen-Kallenberg et al., 2017; Meghani & Harvey, 2016; Jamilah et al., 2021; Smith & Khawaja, 2011; Ulu et al., 2015). As previously mentioned, Nigerian students, influenced by cultural values and societal expectations, often pursue academic success but may face psychological issues due to ineffective coping strategies (Adewunmi, 2015; Andemariam, 2007; Ezeobele et al., 2010). Despite their resilience, they may conceal struggles, fearing social stigma and damage to relationships, which may exacerbate their distress (Iwuno et al., 2023), leading to withdrawal from psychosocial activities and amplifying the challenges of cross-cultural adjustment and acculturative stress.

### 1.2. Acculturative Stress, Loneliness, and Depression

Acculturative stress, linked with feelings of isolation and loneliness, presents challenges in forming social connections within a new cultural environment (Cuijpers et al., 2019; Drake et al., 2016; Koo et al., 2021). Loneliness, a complex emotional state arising from perceived social relationship inadequacies, is prevalent among immigrant populations undergoing cultural adaptation (S. Cacioppo et al., 2015). Numerous studies highlight a strong association between loneliness and depression, suggesting that prolonged social isolation can contribute to clinical depression (J. T. Cacioppo & Hawkley, 2009). In the acculturation context, loneliness may act as a mediator linking acculturative stress and depression, worsening the mental health effects of cultural adjustment challenges.

Interpersonal connectedness and self-identity, counteracting loneliness, are crucial for fulfilling social relationships and defining oneself (Hovey, 2000). Acculturative stress threatens these aspects and can lead to depressive symptoms (M. H. Luz, 2020). Factors associated with adapting to a new country heighten international students’ vulnerability to loneliness, as cultural differences and limited social networks exacerbate feelings of isolation (Meghani & Harvey, 2016). Persistent loneliness among international students may escalate into other mental health issues, such as anxiety and depression (Sawir et al., 2008). Limited social support, reduced social self-confidence, and language challenges may deter international students from seeking assistance and utilizing mental health resources (Jung et al., 2007). Research suggests a bidirectional relationship between acculturative stress and loneliness, with stressors disrupting social networks and cultural dissonance exacerbating loneliness, while loneliness amplifies acculturative stress by heightening feelings of isolation (McDowell et al., 2021; Janssen-Kallenberg et al., 2017). This association may vary across cultural contexts, with collectivist cultures like Nigeria, where social relationships are highly valued, experiencing heightened loneliness when faced with acculturative stressors (Choi, 1997; Y. Jang et al., 2014; Ndika, 2013).

### 1.3. Moderating Role of Perceived Social Support on Acculturative Stress, Loneliness, and Depression

The role of perceived social support in moderating acculturative stress, loneliness, and depression is multifaceted. Social support, comprising instrumental and emotional assistance from sources like family and friends, plays a vital role in aiding international students during their adaptation and mitigating the stress of transition (Lin et al., 1999; Liu et al., 2023). However, it is noted in the literature that perceived social support, when offered without being sought, can potentially have negative consequences on well-being (Y. Kim et al., 2022), especially if it compromises individuals’ autonomy (Baeza-Rivera et al., 2022).

Despite some instances where perceived social support might have adverse implications, it is generally recognized as a key protective factor against negative mental health outcomes. The stress-buffering model suggests that it can reduce the psychological impacts on mental health (Schwarzer & Knoll, 2007). For example, among Chinese college football athletes, high levels of social support were associated with reduced psychological pressure and hopelessness (Liu et al., 2023), and migrant workers in South Korea experienced enhanced psychological well-being due to perceived social support (S. Jang et al., 2023).

Perceived social support not only directly impacts mental health but also moderates the effects of acculturative stress, social isolation, loneliness, and depression among individuals undergoing cultural adaptation (M. H. Luz, 2020). It offers emotional reassurance and validation, alleviating distress and loneliness associated with acculturative stress (Pang et al., 2024). Additionally, it provides practical resources crucial for managing stressors and empowers individuals to cope effectively (Hovey & Magaña, 2000). Feeling supported socially fosters a sense of belonging, facilitating integration into the host culture and resilience against the adverse effects of acculturative stress (Aroian et al., 2017). Culturally congruent social support, aligned with individuals’ values, norms, and beliefs, effectively moderates the impact of acculturative stress on depression (B. S. K. Kim & Omizo, 2005). Such support is perceived as more meaningful, enhancing its protective role against loneliness and depressive symptoms. Ultimately, the sense of belonging and connectedness fostered by perceived social support counteracts feelings of isolation and loneliness, making individuals less susceptible to depression even when facing acculturative stressors (Yeh & Inose, 2003).

### 1.4. Hypotheses and Objectives

**Figure 1:**
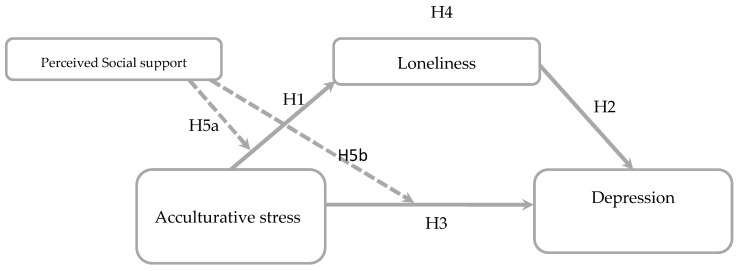
Conceptual model depicting the relationship between acculturative stress and depression, with loneliness as a mediator and perceived social support as a moderator.

This study aims to explore the following: (a) the potential correlation between acculturative stress and depression among Nigerian international university students, (b) whether loneliness may serve as a mediator in the relationship between acculturative stress and depression, and (c) if the direct impact of acculturative stress, loneliness, and depression would be moderated by perceived social support. Drawing upon an integrated model (refer to Figure 1), this study is structured around the following hypotheses:

**Hypothesis 1.**  *There is a direct impact of acculturative stress on loneliness.*

**Hypothesis 2.**  *There is a direct impact of loneliness on depression.*

**Hypothesis 3.**  *Acculturative stress is significantly and positively linked to depression.*

**Hypothesis 4.**  *Loneliness mediates the linkage between acculturative stress and depression.*

**Hypothesis 5a.**  *Perceived social support will moderate the relationship between acculturative stress and loneliness, with higher levels of perceived social support weakening the positive association.*

**Hypothesis 5b.**  *Perceived social support will moderate the relationship between acculturative stress and depression, with higher levels of perceived social support weakening the positive association.*

## 2. Method

### 2.1. Study Design

This study employed a quantitative cross-sectional approach to investigate how loneliness mediates the association between acculturative stress and depression, as well as the moderating influence of perceived social support on the link between acculturative stress and depression among Nigerian international students in Northern Cyprus. Quantitative research designs offer several advantages for addressing social science inquiries. They allow for the examination of large sample sizes, thus enhancing the generalizability of findings. Additionally, they promote objectivity and precision in results, enable the aggregation and analysis of extensive datasets for cross-category and temporal comparisons, and help reduce researcher bias by maintaining a degree of separation from participants and involving unfamiliar individuals (Verhoef & Casebeer, 1997).

### 2.2. Participants Recruitment and Procedures

This study targeted Nigerian international students as the population of interest. Based on a computer-based (G*power3.1) analysis, aiming for a statistical power of 80% with an effect size of 0.15 and an alpha level of 0.05, a sample size of 85 was deemed adequate. In total, 392 Nigerian students were included via convenience sampling conducted online. Inclusion criteria required participants to be registered students, over 18 years old, from Nigeria, and residing in Northern Cyprus. Of these participants, 205 were female (52.3%) and 187 were male (47.7%), predominantly freshmen. This research was approved by the Research Ethics Committee at Girne American University. Prior to data collection, participants provided digital consent, acknowledging the study’s objectives and importance. They were assured of confidentiality, with no personal information recorded, and informed that their responses would be exclusively utilized for research purposes.

Data were collected through web-based questionnaires (Google Forms) from participants across six universities in Northern Cyprus (Girne American University, Final International University, Near East University, University of Kyrenia, Eastern Mediterranean University, and Cyprus International University) at different times, as shown in Table 1.

### 2.3. Measures

#### 2.3.1. Acculturative Stress

Acculturative stress levels were assessed using an adapted 18-item scale, which combined elements from two established measures: the Acculturative Stress Scale for International Students (ASSIS) by Sandhu and Asrabadi (1994) and the Barcelona Immigration Stress Scale by Eiroa-Orosa et al. (2023). This comprehensive scale gauges stress across various aspects of acculturation, encompassing integration challenges, feelings of nostalgia or homesickness, and encountering barriers. Participants indicated their agreement with statements such as “I feel sad living in unfamiliar surroundings” and “I feel uncomfortable adjusting to new foods” on a 5-point Likert scale ranging from “1 (strongly disagree)” to “5 (strongly agree)”. Higher scores denoted heightened levels of acculturative stress.

#### 2.3.2. Depression

Depressive symptoms were evaluated using the depression section of the Patient Health Questionnaire (PHQ-9). This section consists of nine questions drawn from the Diagnostic and Statistical Manual for Mental Disorders—5th edition (DSM-V) criteria for diagnosing depression (Kroenke et al., 2001). Responses are graded on a scale from “0” (not at all) to “3” (nearly every day). An example question from this section is: “Do you often feel bad about yourself, that you are a failure, or have let yourself or your family down?” Total scores range from 0 to 27, with higher scores indicating greater severity of depressive symptoms.

#### 2.3.3. Perceived Social Support

The Perceived Social Support Scale by Zimet et al. (1990) was employed to gauge individuals’ perceptions of social support from various sources. This self-report scale comprises 12 items distributed across three dimensions: family, friends, and other significant sources such as the university. Each dimension consists of four items. Examples include statements like “I can get emotional help and support from my family” for the family dimension, “I can count on my friends when things go wrong” for the friends dimension, and “The international students center on campus is available when I need it” for the other aspect. Participants rate each item on a scale ranging from “1 (very strongly disagree)” to “7 (very strongly agree)”.

#### 2.3.4. Loneliness

Loneliness was evaluated using the UCLA-3 loneliness scale, which is a condensed version of the Revised UCLA Loneliness Scale (UCLA-R). This scale aims to assess both subjective sensations of loneliness and perceptions of social isolation. Comprising three items, the scale covers aspects related to interpersonal bonds, community integration, and individual feelings of disconnection or isolation. For example, a sample question from this scale is “How frequently do you experience feelings of exclusion?”, with response options ranging from “hardly ever” (1) to “often” (3). A score of ≥6 indicates an elevated likelihood of experiencing loneliness (Steptoe et al., 2013).

### 2.4. Statistical Analysis

Following data collection, it was initially entered into version 27 of Statistical Package for the Social Sciences (SPSS). Subsequently, (Partial Least Squares Structural Equation Modeling (PLS-SEM) was utilized to assess the reliability and validity of constructs and to test the research hypotheses.

## 3. Results

### 3.1. Assessment of the Measurement Model

In accordance with Roldán and Sánchez-Franco’s (2012) guidelines, the initial step involved analyzing various metrics such as factorial loads, composite reliability, reliability coefficient rho, Cronbach’s alpha, and average variance extracted (AVE) to assess both convergent and discriminant validity. Factor loading indicates the degree to which an item accurately represents the underlying construct, typically aiming for values above 0.70 as recommended (Vinzi et al., 2010). For reliability assessment, metrics like Cronbach’s alpha (above 0.70 is considered acceptable), composite reliability, and Dijkstra-Henseler’s rho are evaluated, all aiming for scores exceeding 0.70, based on Hair et al. (2016). Convergent validity, measured by AVE, ideally is recommended to surpass the threshold of 0.5 (Hair et al., 2016). Discriminant validity is assessed using heterotrait–monotrait (HTMT), with the recommended value being below 0.85 (Henseler et al., 2015). As shown in Table 2 and Table 3, the values exceed the thresholds recommended by the literature.

### 3.2. Assessment of the Structural Model

**Figure 2:**
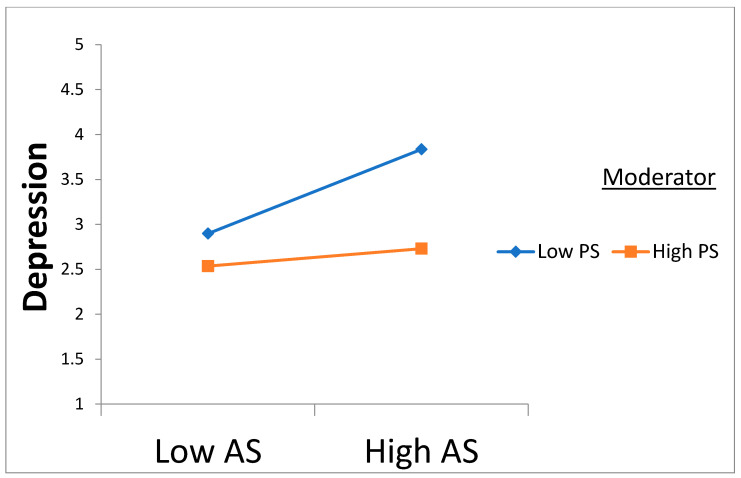
Perceived social support moderating the relationship between acculturative stress and depression.

**Figure 3:**
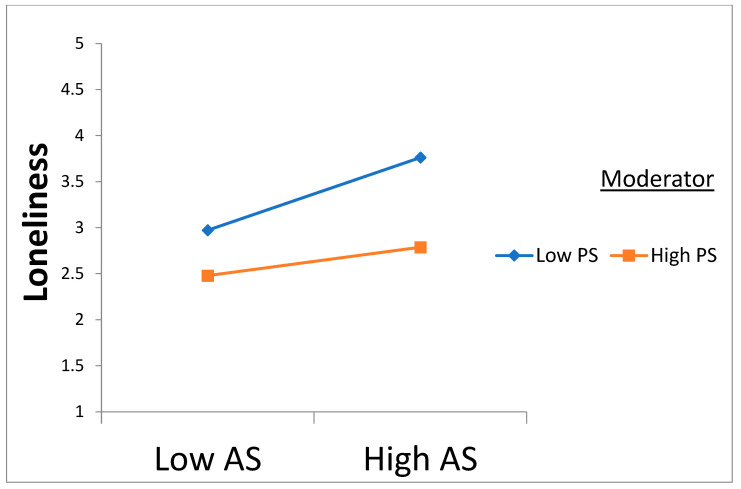
Perceived social support moderating the relationship between acculturative stress and loneliness.

Following the execution of PLS bootstrapping with 5000 samples and 95% confidence intervals using SmartPLS, the overall goodness of fit measures for the PLS model are provided in Table 4. The standardized root mean square residual (SRMR) and Normed Fit Index (NFI) for this investigation are 0.036 and 0.928, respectively, falling within the recommended thresholds proposed by Hu and Bentler (1998) and Henseler et al. (2015) of SRMR below 0.08 and NFI above 0.9. Both R2 and Q2 were evaluated to elucidate the predictive power and relevance. The outcomes of these evaluations are presented in Table 3, meeting the stipulated threshold recommended by Chin (1998) of more than zero. The examination of the structural model affirms that acculturative stress positively impacts the loneliness and depression of Nigerian international students. Loneliness also exerts a positive impact on depression. All direct path coefficients surpass 0.2, exceeding the minimum threshold proposed by Chin (1998). The paths of the model and hypotheses are established as significant, showing the t-values and β coefficients, as detailed in Table 4 and Figure 2 and Figure 3.

Perceived social support dampens the positive relationship between acculturative stress and loneliness and depression.

## 4. Discussion

Drawing on the existing literature, this cross-sectional study investigates the interrelation among acculturative stress, loneliness, and depression in Nigerian international students residing in Northern Cyprus. The primary objective is to construct and assess a model that explores the moderating effect of perceived social support on the association between acculturative stress, loneliness, and depression.

The results confirm a significant correlation between acculturative stress, loneliness, and depression. Notably, loneliness is found to serve as a partial mediator in the relationship between acculturative stress and depression. Moreover, the findings underscore the moderating influence of perceived social support on the dynamics involving acculturative stress, loneliness, and depression.

Consistent with the initial hypothesis (H1), this study identifies acculturative stress as a pivotal factor contributing to feelings of loneliness. Specifically, a positive correlation emerges between acculturative stress and loneliness among Nigerian international students, suggesting that heightened acculturative stress corresponds to increased loneliness. This association aligns with previous research conducted among international students in the United States, indicating that challenges in adapting to a new cultural milieu may foster a sense of isolation and detachment, thereby exacerbating feelings of loneliness (M. H. Luz, 2020).

Previous studies have indicated that international students who share common linguistic and cultural backgrounds tend to experience lower levels of loneliness compared to their counterparts (Zheng et al., 2023). The association between acculturative stress and loneliness can partly be understood through the concept of cultural belonging (Chavajay & Skowronek, 2009). While cultural belonging can serve as a resource for students navigating different cultures during their stay abroad, it can also lead to inner conflicts regarding their sense of belonging. Therefore, when there is a strong sense of cultural attachment and inclusion between two cultures, it can help alleviate loneliness, whereas acculturation stress and cultural conflicts may exacerbate loneliness among international students (Albert, 2021).

For Nigerian students in this particular study, many reported a significant cultural distance between their own culture and that of Northern Cyprus. The substantial differences between their cultural backgrounds and the culture in Northern Cyprus, coupled with the overwhelming associated stress, may contribute to cultural conflicts and an increased sense of loneliness.

The study’s second hypothesis (H2) revealed a direct correlation between loneliness and depression among Nigerian students. Loneliness was found to positively influence depression, indicating that students who feel disconnected and isolated due to loneliness are more susceptible to depressive symptoms. Factors such as social isolation, a lack of meaningful social connections, and feelings of alienation can exacerbate loneliness and, thus, decline in mental health. Moreover, differences in coping strategies and support-seeking behaviors across cultures can further complicate the experience of loneliness and increase vulnerability to depression (Hager et al., 2022). These findings align with a study by McGuinness (2022), which similarly found a significant relationship between loneliness and depression among university students in Ireland.

Cultural factors such as stigma surrounding mental health, reluctance to seek professional help, and norms regarding emotional expression can hinder effective interventions for loneliness and depression. Social factors such as peer support, a sense of belonging, and integration into the host community also play a role in shaping the relationship between loneliness and depression (Oei & Notowidjojo, 1990).

The third hypothesis, H3, demonstrated the direct effect of acculturative stress on depression. The result indicated that Nigerian students facing pressure to adapt to a new language, cultural values, and social norms may experience heightened stress or anxiety, stemming from the disparities between their native culture and the predominant host culture. This stress can significantly impact individuals, potentially leading to depression. Acculturative stress is an inevitable experience for international students as they navigate the psychological challenges of encountering different cultures first-hand. The higher the level of acculturative stress, the more detrimental its effects on mental health (Thomas, 2018). The findings of this study highlighted a significant, positive correlation between acculturative stress and depression, aligning with previous research that identified a similar relationship among both domestic and international students at an international university in Japan (Nguyen et al., 2019). In a similar study, Ma (2017) also observed acculturative stress as a significant predictor of depression among study participants.

The results of the fourth hypothesis (H4) indicated an indirect effect of acculturative stress, partially mediated by loneliness, on depression. This suggests that heightened acculturative stress is linked to increased loneliness, which subsequently predicts higher levels of depression. This finding underscores the significant role of loneliness in translating cultural adaptation stress into depressive symptoms (H. Luz & Thomas, 2023). It confirms that, while acculturative stress directly contributes to depression, part of its impact is also channeled through increased feelings of loneliness among individuals undergoing cultural adaptation. This finding aligns with a study by M. H. Luz (2020), which also found that loneliness partially mediated the relationship between acculturative stress and depression among international students in the US. This implies that loneliness acts as a pathway through which acculturative stress influences depressive symptoms. These findings hold implications for interventions targeting depression among international students, such as implementing social support programs or providing cultural sensitivity training.

The fifth hypotheses, H5a and H5b, proposed that perceived social support plays a substantial moderating role in the correlation between acculturative stress, loneliness, and depression. The outcomes demonstrate that perceived social support significantly influences the relationship between acculturative stress and both loneliness and depression. These results imply that perceived social support acts as a protective barrier, mitigating the impact of elevated acculturative stress on loneliness and depression among the participants. Results from the simple slopes figures also indicate that high and low levels of perceived social support have significant effects on loneliness and depression, with the impact being more pronounced at lower levels of support. This supports hypotheses H5a and H5b, showing that perceived social support can either alleviate or worsen the effects of acculturative stress on mental health outcomes. Specifically, higher levels of perceived social support weaken the relationship between acculturative stress and feelings of loneliness and depression (Baeza-Rivera et al., 2022). This aligns with a study by Y. Kim et al. (2022), which found that individuals with strong perceived social support, especially emotional support, were less affected by acculturative stress in terms of depressive symptoms. Emotional support, such as empathy and reassurance, plays a vital role in mitigating the negative effects of acculturative stress on depression (Oppedal & Idsoe, 2015). In essence, the quality of support, rather than just its presence, is essential in buffering the psychological effects of cultural adaptation challenges. Furthermore, cultural factors may influence how perceived social support affects acculturative stress. Cultural values shape individuals’ perceptions of support and its impact on mental health outcomes (Zhou et al., 2022). Collectivist cultures, which emphasize interdependence and familial support, may rely more on family networks, whereas individualistic cultures may lean towards peer or community support (Jacobson, 1987). Consequently, the effectiveness of perceived social support in alleviating acculturative stress may differ across cultural contexts.

Overall, the findings highlight the need for holistic interventions to the needs of international students traversing the murky landscape of adjustment in an unfamiliar academic environment. Students’ health care providers should integrate strategies that address multiple ecosystem elements that include educational policies, family engagement, and accessible counselling services to boost overall well-being of international students.

### Limitation and Future Study

This study may exhibit biases because it relies solely on data collected through self-report questionnaires. Additionally, the findings derived from the current sample, which treats Nigeria as a singular entity without acknowledging its diverse ethnic groups and cultural variations, might not accurately represent attitudes toward acculturation and mental health. Future researchers might consider replicating this study with samples stratified by ethnic groups to enable more comprehensive and precise generalizations. Cross-sectional studies are limited in their ability to establish causal relationships between acculturative stress, perceived social support, loneliness, and depression, highlighting the necessity for long-term longitudinal research to validate the associations between these variables. This study did not assess the impact of demographic variables such as gender, relationship status, religiosity, length of stay, previous travel experience, and cultural distance on acculturative stress and mental health. Therefore, future investigations should explore these factors to confirm their relationship with acculturative stress and mental health.

## 5. Conclusions

The findings of this study are noteworthy as they validate the phenomenon of acculturative stress and depression among Nigerian international students, who often face challenges in adapting to a new cultural and academic environment. The acculturation process and mental health of international students are significant phenomena with implications for students, universities, and host countries. Therefore, understanding the interrelationships among variables influencing mental health provides essential insights to guide policy-making efforts aimed at promoting the integration and well-being of international students in host countries. Institutions should create a targeted holistic approach that addresses diverse ecosystem elements with strategic provision of institutional support services, promotion of cross-cultural interaction campaigns, and inclusive social environment. Future studies should explore longitudinal approaches, possibly for the duration of their studies, for a better understanding of the dynamic interplay of acculturative stress and potential mental health problems. Additionally, investigating the role of other potential mediators such as coping mechanisms or moderators like cultural identity may provide a valuable insight to understanding those relationships. Expanding the research to other nationalities, with diverse cultural background, would enrich the literature and provide comprehensive strategies to ameliorate acculturative stress and mitigate its effect on mental health outcomes.

## Figures and Tables

**Table 1:** Frequency distribution of participants’ demographic characteristics.

Variables	Categories	N/(%)	Total N
Gender	Male	187 (47.7)	392
	Female	205 (52.3)	
Education	Bachelor	248 (63.3)	392
	Masters	127 (32.4)	
	Doctorate	17 (4.3)	
Year	1st	212 (54.1)	392
	2nd	67 (17.1)	
	3rd	44 (11.2)	
	4th	69 (17.6)	
Marital Status	Married	66 (16.8)	392
	unmarried	326 (83.2)	
Friends with locals	Yes	242 (61.7)	392
	No	150 (38.3)	
Cultural distance	Extremely different	10 (2.6)	392
	A lot of difference	12 (3.1)	
	Some difference	44 (11.2)	
	A little different	24 (6.1)	
	No different	24 (6.1)	
Turkish Language proficiency	Not at all	24 (6.1)	392
	poor	163 (41.6)	
	Fair	85 (21.7)	
	Good	82 (20.9)	
	Very good	38 (9.7)	392

**Table 2:** Reliability, factor loadings, and convergent validity.

Variables	Items	Outer Loadings	Cronbach Alpha	Rho A	CR	AVE
Acculturative stress	AS1	0.914	0.864	0.866	0.909	0.676
	AS2	0.741				
	AS3	0.802				
	AS4	0.913				
	AS5	0.785				
	AS6	0.877				
	AS7	0.781				
	AS8	0.741				
	AS9	0.745				
	AS10	0.941				
	AS11	0.697				
	AS12	0.741				
	AS13	0.842				
	AS14	0.913				
	AS15	0.741				
	AS16	0.802				
	AS17	0.813				
	AS18	0.940				
Perceived Social Support	PS1	0.741	0.818	0.836	0.872	0.740
	PS2	0.742				
	PS3	0.913				
	PS4	0.781				
	PS5	0.941				
	PS6	0.745				
	PS7	0.922				
	PS8	0.914				
	PS9	0.981				
	PS10	0.841				
	PS11	0.947				
	PS12	0.799				
Depression	DP1	0.912	0.866	0.883	0.897	0.759
	DP2	0.917				
	DP3	0.891				
	DP4	0.902				
	DP5	0.814				
	DP6	0.817				
	DP7	0.816				
	DP8	0.901				
	DP9	0.862				
Loneliness	LO1	0.796	0.755	0.779	0.836	0.638
	LO2	0.819				
	LO3	0.781				

**Table 3:** Heterotrait–monotrait ration (HTMT).

	AS	DP	LO	PS
AS	—			
DP	0.735	—		
LO	0.675	0.618	—	
PS	0.401	0.629	0.558	—

**Table 4:** Results of the hypothesis testing.

Paths	Combinations	β	t	F^2^	Decision
Direct effects					
H_1_	AS→LO	0.421	9.46 ***	0.042	Supported
H_2_	LO→DP	0.434	9.65 ***	0.045	Supported
H_3_	AS→DP	0.514	11.26 ***	0.155	Supported
Mediation					
H_4_	AS→LO→DP	0.187	3.294 **	0.032	Supported
Moderation					
H_5a_	AS*PS→LO	−0.120	−2.332 *	0.023	Supported
H_5b_	AS*PS→DP	−0.186	−2.738 *	0.026	Supported
	R^2^_LO_ = 0.580/Q^2^_LO_ = 0.337 R^2^_DP_ = 0.838/Q^2^_LO_ = 0.416 SRMR = 0.036; NFI = 0.928				

AS = acculturative stress, LO = loneliness, DP = depression, PS = perceived social support. * *p* < 0.10; ** *p* < 0.05; and *** *p* < 0.01.

## Data Availability

The original contributions presented in this study are included in the article. Further inquiries can be directed to the corresponding author.
